# PDIL1-2 can indirectly and negatively regulate expression of the *AGPL1* gene in bread wheat

**DOI:** 10.1186/s40659-019-0263-2

**Published:** 2019-11-07

**Authors:** Jie Dong, Yongxing Zheng, Yihan Fu, Jinxi Wang, Shasha Yuan, Yonghua Wang, Qidi Zhu, Xingqi Ou, Gezi Li, Guozhang Kang

**Affiliations:** 1 The National Key Laboratory of Wheat and Maize Crop Science, Henan Agricultural University, #15 Longzihu College District, Zhengzhou, 450046 China; 2 The National Engineering Research Center for Wheat, Henan Agricultural University, #63 Nongye Road, Zhengzhou, 450046 Henan China; 3 The School of Science and Technology, Henan Institute of Science and Technology, Xinxiang, 453003 China

**Keywords:** AGPase, BSMV-VIGS, Protein disulfide isomerase, Starch biosynthesis, *Triticum aestivum* L., Yeast one-hybrid

## Abstract

**Background:**

ADP-glucose pyrophosphorylase (AGPase), the key enzyme in plant starch biosynthesis, is a heterotetramer composed of two identical large subunits and two identical small subunits. AGPase has plastidial and cytosolic isoforms in higher plants, whereas it is mainly detected in the cytosol of grain endosperms in cereal crops. Our previous results have shown that the expression of the *TaAGPL1* gene, encoding the cytosolic large subunit of wheat AGPase, temporally coincides with the rate of starch accumulation and that its overexpression dramatically increases wheat AGPase activity and the rate of starch accumulation, suggesting an important role.

**Methods:**

In this study, we performed yeast one-hybrid screening using the promoter of the *TaAGPL1* gene as bait and a wheat grain cDNA library as prey to screen out the upstream regulators of *TaAGPL1* gene. And the barley stripe mosaic virus-induced gene-silencing (BSMV-VIGS) method was used to verify the functional characterization of the identified regulators in starch biosynthesis.

**Results:**

Disulfide isomerase 1-2 protein (TaPDIL1-2) was screened out, and its binding to the *TaAGPL1*-*1D* promoter was further verified using another yeast one-hybrid screen. Transiently silenced wheat plants of the *TaPDIL1*-*2* gene were obtained by using BSMV-VIGS method under field conditions. In grains of BSMV-VIGS-TaPDIL1-2-silenced wheat plants, the *TaAGPL1* gene transcription levels, grain starch contents, and 1000-kernel weight also significantly increased.

**Conclusions:**

As important chaperones involved in oxidative protein folding, PDIL proteins have been reported to form hetero-dimers with some transcription factors, and thus, our results suggested that TaPDIL1-2 protein could indirectly and negatively regulate the expression of the *TaAGPL1* gene and function in starch biosynthesis.

## Background

Bread wheat is one of the most important cereal crops, accounting for approximately 20% of the daily calorie consumption worldwide, and starch is composed of approximately 70% wheat grains and is the most abundant storage carbohydrate in this organ [1]. AGPase uses the substrates glucose-1-phosphate (G-1-P) and ATP to produce pyrophosphate and ADP-glucose, the starch precursor, and is thought to be the key enzyme in starch synthesis [2]. In plants, AGPase is a heterotetramer composed of two large/modulatory subunits (LSU, or AGPL) and two small/catalytic subunits (SSU or AGPS) [3]. According to its cellular localization, AGPases have cytosolic and plastidial isoforms and these two types function independently to produce starch in cytosol and plastid, respectively. Thus, in plant cells, there are four types of AGPase subunits: cytosolic SSU, plastidial SSU, cytosolic LSU and plastidial LSU [4]. In the plant cells of most dicotyledonous plants, AGPase is active exclusively in plastids, but it is mainly present in the cytosol in the grain endosperm of the grass family, demonstrating approximately 85%, 90%, and 95% of the total AGPase activity in barley, rice, and maize, respectively [5–7], suggesting a crucial role of cytosolic AGPase in starch synthesis in these species.

The number of AGPase subunits is species-specific. For example, four AGPase large subunits (AGPL1, AGPL2, AGPL3, and AGPL4) have been identified in rice and maize, only two subunits, AGPL1 and AGPL2, are present in barley, and the extents to which they play roles in starch biosynthesis are different [8, 9]. Our previous studies have demonstrated that the expression of the *TaAGPL1* gene encoding the cytosolic large subunit of wheat AGPase shows similar changes to the rate of starch accumulation, and its transcripts in wheat grains are significantly and positively related to the activity of AGPase and the accumulation rate of grain starch [10]. Moreover, overexpression of *TaAGPL1* markedly enhanced AGPase activity and the rate of starch accumulation in wheat grains [11]. These data demonstrate that *TaAGPL1* plays an important role in AGPase enzyme and starch biosynthesis in bread wheat and could have potential applications in wheat breeding to develop high-yield wheat cultivars [11]. However, the upstream regulatory mechanism of the *TaAGPL1* gene family remains elusive.

Here we used the *TaAGPL1* promoter as bait to screen an immature wheat grain cDNA library using a yeast one-hybrid (Y1H) assay to isolate upstream regulators. Among the identified proteins, one protein disulfide isomerase 1-2 (TaPDIL1-2) was selected to evaluate its binding to the *TaAGPL1* promoter and test its potential function in regulating *TaAGPL1* expression and starch biosynthesis.

## Methods

### Plant materials

Two semi-winter bread wheat cultivars, Zhoumai 18 and Bainong 207, characterized with high yield potentials, have widely been planted in the Yellow and Huai Valleys Winter Wheat Zone. The former was used in our previous studies [10, 12], and in this study, its leaf DNA was extracted to isolate the *TaAGPL1*-*1D* promoter and its grains were sampled for endosperm cDNA library synthesis and for detection of the *TaAGPL1*-*1D* promoter activity. The latter is relatively sensitive to plant viruses and was used for the BSMV-VIGS assay [13].

### GUS activity of the *TaAGPL1*-*1D* gene assay

The sequence of *TaAGPL1*-*1D* gene was used as a query to search for its upstream promoter sequence in the International Wheat Genome Sequencing Consortium (IWGSC) database. A 1280-bp fragment was retrieved and used as its promoter sequence. A pair of primers were designed to amplify this fragment from cv. Zhoumai 18 (Additional file 1: Table S1), and then a series of nested 5′ deletions of this promoter were inserted into the pCAMBIA1301 vector containing the CaMV35S promoter to drive expression of the *GUS* gene, forming the Pro-1: GUS (− 1269 bp to **+ **11 bp), Pro-2: GUS (− 870 bp to **+ **11 bp), and Pro-3: GUS (− 483 bp to **+**11 bp) recombinant vectors, respectively. GUS activity of *TaAGPL1*-*1D* was assessed using a transient transformation system in *N. benthamiana* plants or immature wheat grains. In tobacco plants, the recombinant plasmids of Pro-1: GUS, Pro-2: GUS, and Pro-3: GUS were transferred into the *Agrobacterium* EHA105 lines, and then the EHA105 lines were coinfiltrated into the *N. benthamiana* leaves [14]. For wheat grains, immature grains sampled at 20 days after anthesis were used to qualitatively and quantitatively determine the *TaAGPL1*-*1D* promoter activity using gene gun-mediated transient transformation protocol [15]. The recombinant plasmids of Pro-1: GUS, Pro-2: GUS, and Pro-3: GUS were transfected using the PDS-1000/He Particle Delivery SystemTM (Bio-Rad, Hercules, CA, USA). Stained samples were photographed using a Canon Digital Camera EOS 70 D (Canon Co., Ltd., Zhuhai, China). Quantitative GUS activity was subsequently measured [16].

### Identification of TaPDIL1-2 protein by Y1H screening

Approximately 2 mg of high-quality total RNA from wheat endosperm sampled at 5, 10, 15, 20, 25, 30, and 35 days after anthesis was mixed and extracted with TRIzol reagent (Invitrogen, Carlsbad, CA, USA). Wheat endosperm cDNA library was constructed using the Advantage 2 PCR Kit [Cat No. 639206, TaKaRa Biotechnology Co., Ltd., Dalian, China]. The *TaAGPL1*-*1D* promoter was used to construct the bait vector (pAbAi-TaAGPL1). The pAbAi-TaAGPL1 vector was then linearized and integrated into the genome of yeast strain Y1HGold. The bait strain Y1HGold/TaAGPL1-pro-pAbAi survived on SD/-Ura medium. The plasmids of the wheat endosperm cDNA library were next transformed into competent cells of the bait strain, and the transformed yeast cells grew on plates of SD/-Leu, -Ura, +AbA^100^ at 30 °C for 2–4 days. The positive yeast colonies were isolated and confirmed by PCR amplification with 5′ T7-F and 3′ AD-R primers (Additional file 1: Table S1).

The interaction between TaPDIL1-2 protein and *TaAGPL1*-*1D* promoter was measured using another Y1H assay. Three fragments (Pro-1, 1280 bp, Pro-2, 881 bp and Pro-3, 494 bp) of the *TaAGPL1*-*1D* promoter were separately ligated into the pAbAi vector. ORF (open reading frame) sequence of *TaPDIL1*-*2* gene was cloned and then ligated into the pGADT7 vector to produce the prey vector. Two concentrations (0 and 100 ng/mL) of AbA were used, and the *TaPDIL1*-*2* prey vector was transformed into yeast Y1HGold strains containing the Bait-Reporter vectors. pGADT7-TaPDIL1-2 and pAbAi-TaAGPL1 promoters were co-transformed into the Y1HGold yeast strains. The transformed cells were grown on SD/-Leu, -Ura selective medium with 100 ng/mL AbA at 30 °C for 2–4 days.

Transcriptional expression data for the *TaAGPL1*-*1D* gene in grain, spike, leaf/stem at reproductive and vegetative stages were obtained from the expVIP database and then viewed as a heat map using the HemI-1.0 tool (Heatmap illustrator) [17].

### BSMV-VIGS experiment

To alleviate functional complementation and allow complete silencing, we selected a conserved cDNA fragment (190 bp) of the *TaPDIL1*-*2* gene, three homoeologs of which shared 99.1% similarity, possibly enabling simultaneous silencing using the BSMV-VIGS method. The primers used for the BSMV-TaPDIL1-2 vector are shown in Additional file 1: Table S1. Viral vector construction, viral RNA transcription, and viral inoculation of BSMV-VIGS vectors were performed as described in our previous studies [13, 18]. BSMV-GFP-inoculated wheat seedlings were used as the negative control, and the appearance of chlorosis on the inoculated wheat spikes confirmed the successful BSMV inoculation [19]. In the field, BSMV inoculation was performed in the spike of wheat plants at the heading stage, and a total of 142 and 167 spikes were inoculated with BSMV-TaPDIL1-2 or BSMV-GFP, respectively, using a 20-µL transcript mixture for each spike.

Transcription levels of the *TaPDIL1*-*2* gene in the wheat grains at 19, 24, 29, and 34 days after anthesis were measured using the quantitative real-time PCR (qPCR) [20]. The wheat *Actin* and glyceraldehyde 3-phosphate dehydrogenase (*GAPDH*) (GenBank accession no. AB181991 and EF592180) genes were used as internal controls.

### Statistical analysis

One-way analysis of variance (ANOVA) was performed using SPSS version 17.0 software. Data are the mean ± standard deviation (SD) of at least three independent experiments. A multiple range test was used to compare the mean values at the *P* < 0.05 level.

## Results and discussion

### Isolation of the TaAGPL1 promoter

Bread wheat genome contains three closely related, yet distinct, subgenomes (AABBDD) and three homoeologs, with over 95% sequence identity in their coding regions for the majority of genes [17]. In bread wheat, rice and maize, *AGPL1* gene transcripts showed high levels in endosperm, whereas they were not detected in leaves or other organs [7–9, 12], suggesting that the promoter could be endosperm-specific. The cDNA sequence of the *TaAGPL1* gene, which was highly expressed in endosperm during the grain filling period in our previous study [10], was searched against the recently published IWGSC databases (RefSeq v1.0) [21], and a high level (100% identities) to a chromosome-located contig (TraesCS1D01G427400.1) was found, suggesting its localization on 1D chromosome.

**Fig. 1:**
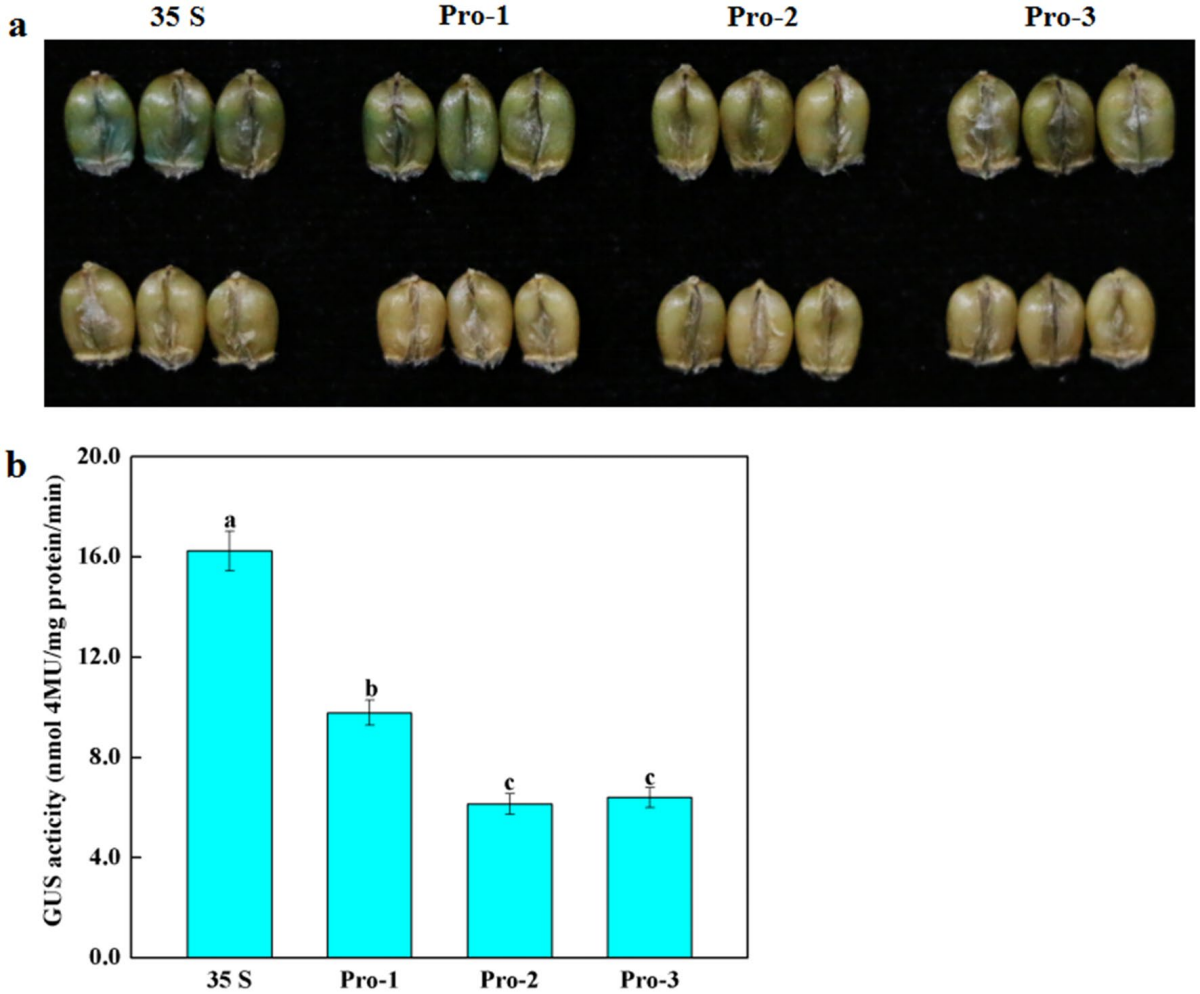
Qualitative and quantitative detection of the *TaAGPL1* promoter-driven GUS activity in the wheat grains. **a** Qualitative staining observation on GUS driven by three fragments of the TaAGPL1 promoter. Upper panel, Pro-1, Pro-2, and Pro-3 represent 1280 bp (− 1269 bp to + 11 bp), 881 bp (− 870 bp to + 11 bp), and 494 bp (− 483 bp to + 11 bp) of the TaAGPL1 promoter, respectively; 35 S represents CaMV35S promoter; Lower panel, negative controls. **b** Quantitative detection on GUS activities driven by three different fragments of *TaAGPL1* promoter or 35S promoter. Each value is mean ± standard deviation of three biological replicates, and different letters indicate significant differences (*P* < 0.05) relative to control by using one-way ANOVA of Duncan’s multiple range test

The sequence of the *TaAGPL1*-*1D* gene was used as a query to search its upstream promoter sequence in the IWGSC database, and a 1280-bp fragment (− 1269 bp to **+ **11 bp) was retrieved and used to design the primer pairs to amplify its promoter sequence. The amplified *TaAGPL1*-*1D* promoter demonstrated high similarity (99.8%) to the retrieved IWGSC sequence (Additional file 2: Fig. S1). Using the classic transient transformation system [14], we did not detect histochemical GUS activity of the *TaAGPL1*-*1D* promoter in the leaves of transiently transformed-*N*. *benthamiana* plants (Additional file 3: Fig. S2), possibly due to its endosperm-specific expression profile. Alternatively, a gene gun-mediated transient transformation protocol for wheat grains was used to detect *TaAGPL1*-*1D* promoter activity [16]. Our experiment showed that the *TaAGPL1*-*1D* promoter qualitatively and quantitatively drove expression of the *GUS* gene (Fig. 1), arguing that this promoter contains *cis*-acting elements that regulate *TaAGPL1*-*1D* gene expression in wheat grains.

### The binding between *TaPDIL1*-*2* gene and *TaAGPL1* promoter

Wheat grains were sampled at 5, 10, 15, 20, 25, 30, and 35 days after anthesis, endosperm was reserved and other organs were removed. The mRNA isolated from the sampled endosperm was used as template to synthesize the full-length double-stranded complementary DNA and together with the linearized pGADT7-Rec vector was co-transformed into yeast strain Y1HGold cells for directional combination through homologous recombination. The combined plasmids were extracted and subsequently transformed into *E*. *coli* strain stellar cells. The total capacity was 1.08 ± 0.3 × 10^6^ cfu with more than 77.9% inserted fragments greater than 750 bp (Additional file 4: Fig. S3), which was suitable for yeast screening.

**Fig. 2:**
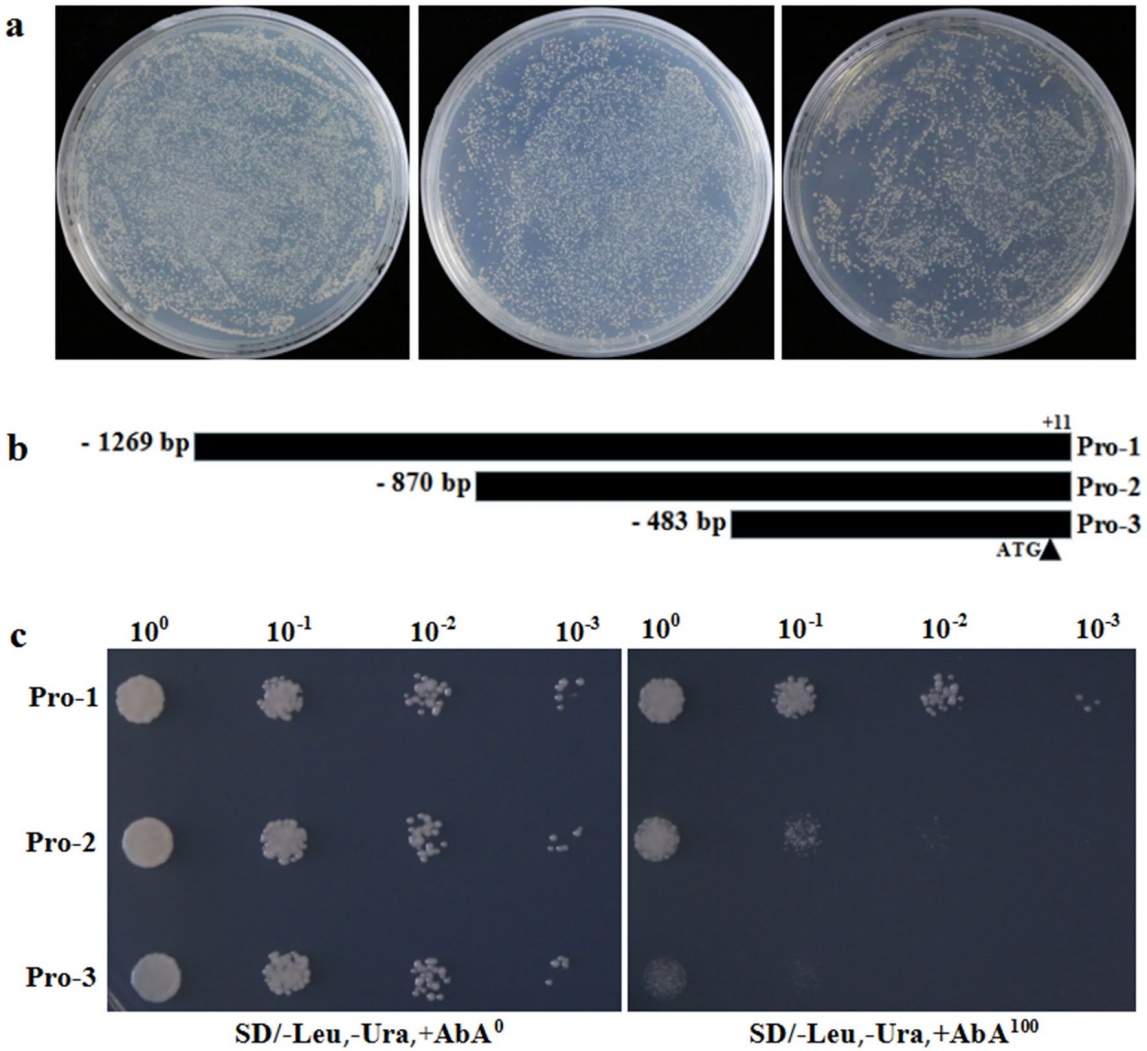
Y1H assay between TaPDIL1-2 protein and *TaAGPL1*-*1D* promoter. **a** cDNA library screen of *TaAGPL1*-*1D* promoter in yeast cells; **b** different fragments (Pro-1, 1280 bp, Pro-2, 881 bp, and Pro-3, 494 bp) of *TaAGPL1*-*1D* promoter using Y1H assay; **c** yeast cells transformed with both the bait vectors containing the above three promoter fragments fused to a pAbAi reporter gene, and a prey vector containing TaPDIL1-2 fused to a pGADT7 activation domain. Yeast cells were grown in liquid media to an OD600 of 0.02, and diluted in a 10 × dilution series (from 100 to 10^−3^). Each dilution 5 μL was spotted on media selecting for plasmids (SD/-Leu, -Ura), supplemented with 0 and 100 ng/mL aureobasidin A (AbA)

Using the above-amplified *TaAGPL1*-*1D* promoter as bait and wheat endosperm cDNA library as prey, we performed Y1H screening (Fig. 2a), and identified 39 positive clones, which were subsequently sequenced and functionally annotated (Additional file 5: Table S2). Of these clones, the *TaPDIL1*-*2* gene was screened out. The complete open reading frame (ORF, 1539 bp) of the *TaPDIL1*-*2* gene was subsequently cloned (Additional file 6: Fig. S4), and its prey vector was constructed and transformed into the bait strain Y1HGold containing three fragments (1280 bp, 881 bp, or 494 bp) of the *TaAGPL1*-*1D* promoter (Fig. 2b), respectively, to perform the second Y1H screening with 0 or 100 ng/mL AbA. Our experiment showed that the transformed yeast cells containing Y1HGold/Pro-1 (− 1269 bp to **+ **11 bp) grew optimally in SD/-Leu, -Ura, +AbA^100^ medium, followed by Y1HGold/Pro-2 (− 870 bp to **+ **11 bp) and Y1HGold/Pro-3 (− 483 bp to **+ **11 bp) (Fig. 2c). These data confirmed the binding between TaPDIL1-2 protein and *TaAGPL1*-*1D* promoter, with the potential binding region located in the promoter between − 483 and − 1269 bp.

### Characterization and expression of TaPDIL1-2

**Fig. 3:**
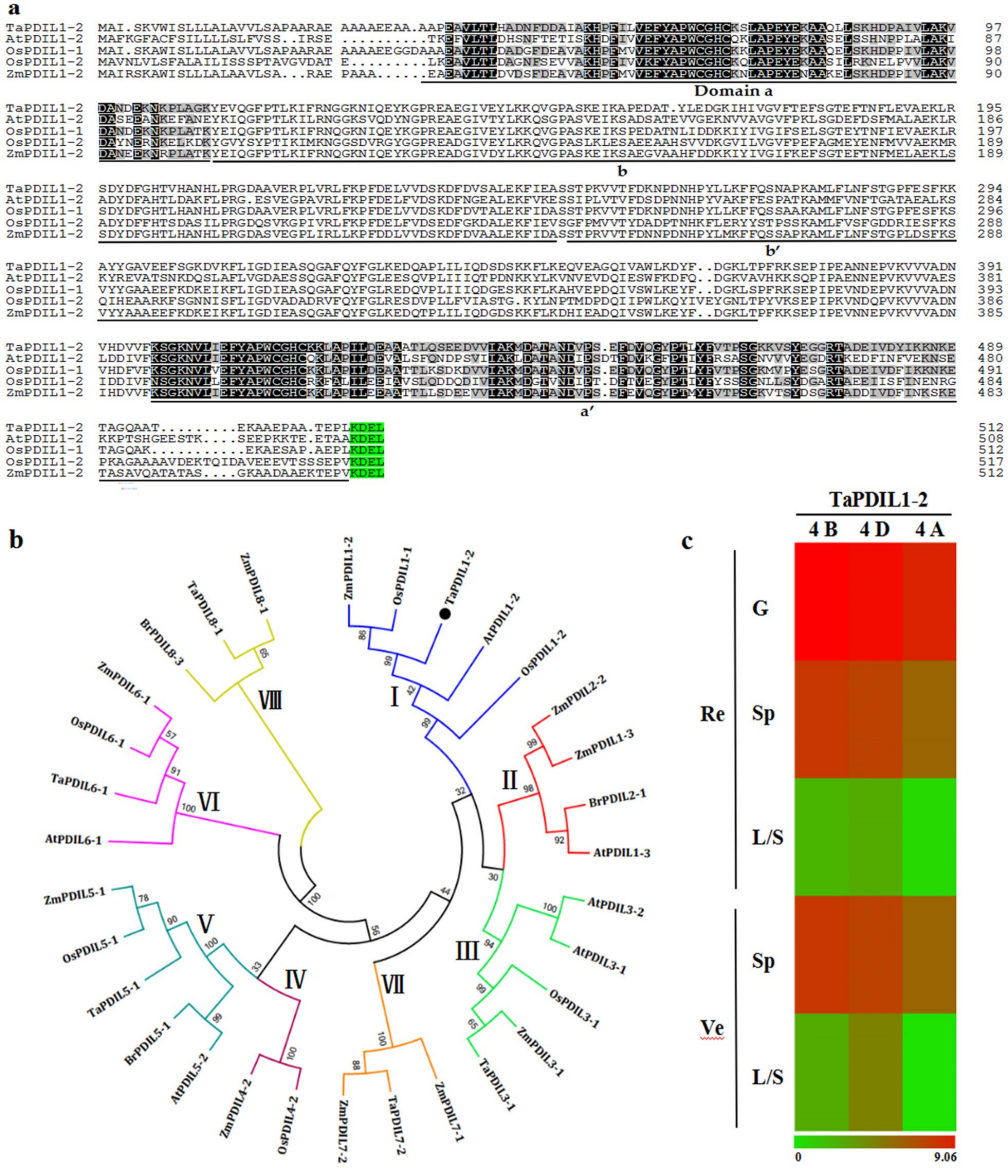
Domains, phylogenetic relationships and expression profiles of TaPDIL1-2. **a** Putative amino acid sequence of the protein encoded by the isolated *TaPDIL1*-*2* in this study. **a**, **a**′, **b**, **b**′ Represent two redox active site CGHC and two inactive domains, respectively, and they are underlined with black. C-terminal KDEL signal sequence is marked with green color; **b** phylogenetic analysis of plant PDILs. The phylogenetic tree was constructed using neighbor-joining phylogeny of MEGA 5.0 with default parameter; **c** In silico expression profiling of *TaPDIL1*-*2* homoeologs in different organs at different growth stages of cv. Chinese Spring wheat. The expression data were generated from the expVIP database. L/S, leaf/stem; Sp, spike; G, grain; Ve, vegetative stage; Re, reproductive stage; 4B, 4D, and 4A represent *TaPDIL1*-*2* 4B, 4D and 4A homoeologs, respectively

By searching the IWGSC databases, the isolated *TaPDIL1*-*2* gene was localized to 4BS, and its ORF encoded a 512-amino-acid protein of 56.43 kDa with a predicted pI of 5.03. The deduced TaPDIL1-2 protein contained 4 classical thioredoxin domains, including 2 redox CGHC active sites (a and a′), 2 inactive domains (b and b′), and 1 C-terminal KDEL signal sequence (KDEL), which is a classcial endoplasmic reticulun (ER)-retention signal supporting ER-localization (Fig. 3a). Phylogenetic analysis indicated that the thioredoxin domain of TaPDIL1-2 shared more than 75.7% identity with those of AtPDIL1-2, OsPDIL1-1, OsPDIL1-2 and ZmPDIL1-2, belonging to one sub-branch of group I in the PDIL subfamily (Fig. 3b). Some PDIL proteins have been identified and functionally characterized in the wheat genome [22]. To our knowledge, however, there have been no reports regarding TaPDIL1-2 or its orthologous genes, and the amino acid sequence of TaPDIL1-2 had low similarities (< 24.3%) to other wheat PDIL proteins (Fig. 3b).

At the transcriptional level, the in silico expression data revealed three homoeologs of the *TaPDIL1*-*2* gene that were more highly expressed during the reproductive stage than the vegetative stages, and during the reproductive stage, their expression level were higher in the endosperm/grain and spikes than in stem and leaf (Fig. 3c). The bread wheat endosperm mainly accumulates starch (≥ 70%) and protein (10–14%), forming the bulk of the grain. These findings suggest that TaPDIL1-2 homoeologs can function in starch biosynthesis in wheat grain.

### Negative regulation of the *TaPDIL1*-*2* gene in wheat starch biosynthesis

Gene function in higher plants is often explored through the use transgenic and mutational assays. In bread wheat, however, the multiple copy insertions, low transformation efficiency, cultivar specificity, time consumption, and high cost of transgenic approaches have greatly limited gene function studies in this species [23]. The functional redundancy among homoeologs in this species also causes some difficulties in terms of generating null mutants [24]. BSMV-VIGS method facilitates the rapid generation of gene knockdown phenotypes in polyploid species because plant transformation is not required, accelerating the characterization of target genes [25]. In this study, this method was used to evaluate the function of the *TaPDIL1*-*2* gene. We constructed BSMV-TaPDIL1-2 and BSMV-GFP vectors, and the latter was used as the control. To prevent functional complementation and allow complete silencing, we selected a conserved cDNA fragment (190 bp) of the *TaPDIL1*-*2* gene, three homoeologs of which shared 99.1% similarity (Additional file 7: Fig. S5), potentially enabling the simultaneous silencing of its three homoeologs using the BSMV-VIGS method.

**Fig. 4:**
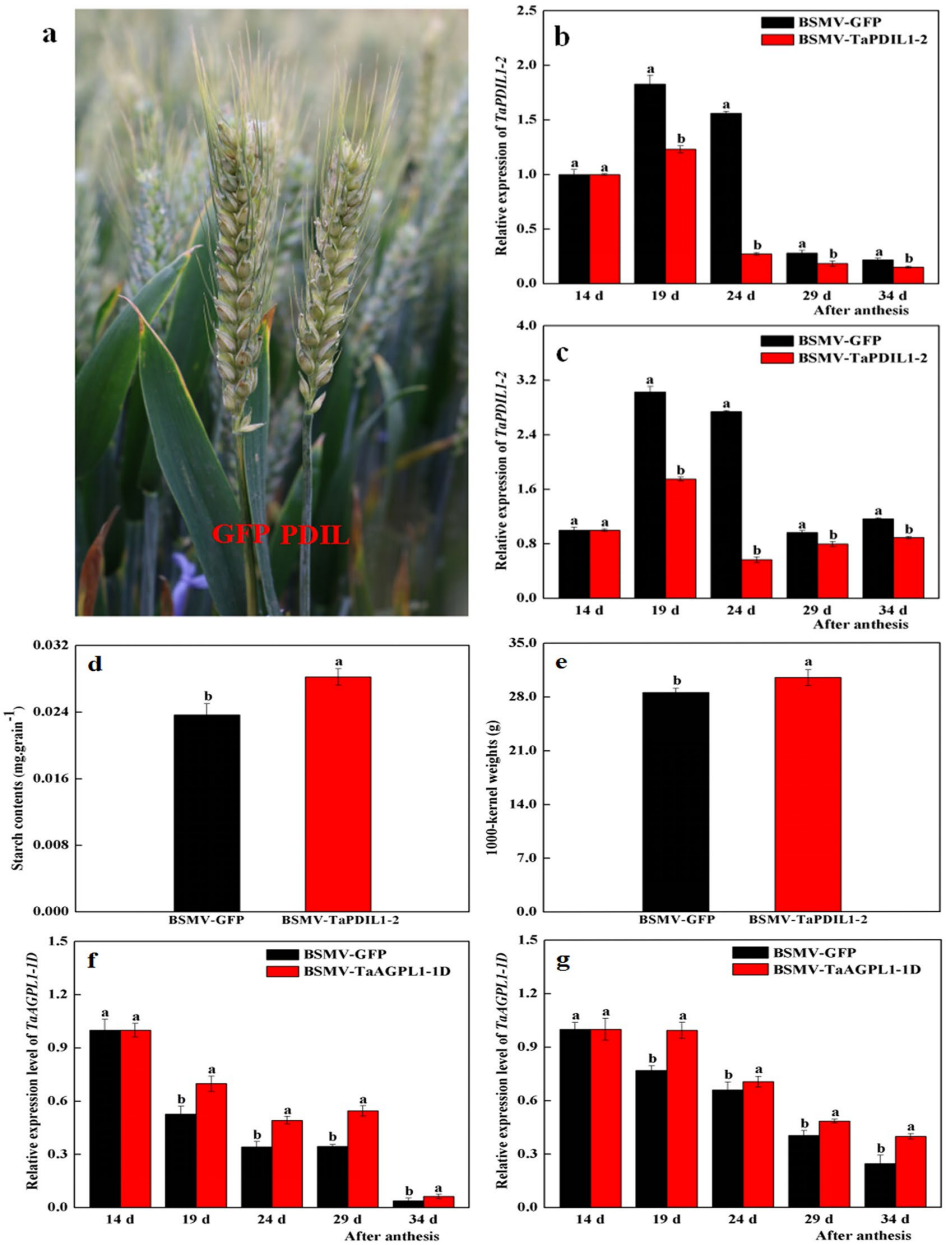
Phenotypes and its transcription levels of *TaPDIL1*-*2*-silencing wheat plants. Grain starch contents and 1000-kernel weight of BSMV-TaPDIL1-2-silenced wheat plants, and transcription levels of *TaAGPL1*-*1D* gene. **a** Phenotypes of BSMV-TaPDIL1-2-, and BSVM-GFP-inoculated wheat spikes at 19 days after anthesis, respectively; **b**, **c** transcriptional levels of the *TaPDIL1*-*2* gene at 19, 24, 29, and 34 days after anthesis detected by using the qPCR method with the wheat *Actin* (**b**) and *GAPDH* (**c**) genes as two internal controls; d and e, grain starch contents and 1000-kernel weight, respectively; **f**, **g** transcription levels of the *TaAGPL1-1D* genes in inoculated wheat grains at 19, 24, 29, and 34 days after anthesis with *Actin* (**f**) and *GAPDH* (**g**) genes as two internal controls, respectively. Each value is mean ± standard deviation of three biological replicates, and different letters indicate significant differences (*P* < 0.05) relative to control by using one-way ANOVA of Duncan’s multiple range test

In previous studies, the BSMV-VIGS method has mostly been applied to explore the function of candidate genes of cereal crops in controlled conditions considering responses to single or several environmental factors [26–28]. However, there are some differences (e.g. stronger plants, more yields) in growth and phenotypes of most higher plants including important crops grown between field conditions with multiple and variable factors (light, temperature, etc.) and under the controlled conditions [26]. Thus, field experiments can more efficiently explore the function of the target genes. By using arch plastic sheds combined with water-sprayed spikes, a few efficient and convenient approaches were designed in our previous study for application of the BSMV-VIGS method to wheat plants under field conditions [13]. In the present study, BSMV-TaPDIL1-2 and BSMV-GFP vectors were separately used to inoculate 142 and 167 wheat spikes at anthesis under field conditions. We observed that BSMV-VIGS-induced chlorosis occurred on inoculated-spikes at 19 days after anthesis, gradually extended throughout the entire spike, and persisted until the mature stage (Fig. 4a), suggesting that these two virus vectors were successfully inoculated into the wheat spikes.

At 19, 24, 29, and 34 days after anthesis, the transcription levels of the *TaPDIL1*-*2* gene were determined by using qPCR, and the *β*-actin and *GAPDH* genes were used as two internal controls. Our results showed that transcription levels of the *TaPDIL1*-*2* gene in grains of BSMV-TaPDIL1-2-inoculated spikes were markedly decreased by 42.2–79.2% (Fig. 4b, c), similar to previous studies [13, 29]. The starch contents and 1000-kernel weight in grains of BSMV-TaPDIL1-2-inoculated wheat spikes were increased by 16.6% and 6.8%, respectively (Fig. 4d, e), demonstrating that *TaPDIL1*-*2* silencing caused enhanced starch biosynthesis in the wheat endosperm. Thus, *TaPDIL1*-*2* might act as a negative regulator of starch biosynthesis. Similarly, the transcription levels of the *TaAGPL1*-*1D* gene in the endosperm of BSMV-TaPDIL1-2-infected wheat spikes at the four sampling time points increased remarkably (Fig. 4f, g). Because our previous studies have confirmed the important role of the *TaAGPL1* gene in starch biosynthesis in wheat grain [11], we inferred that the *TaPDIL1*-*2* gene could play a crucial role in wheat starch biosynthesis by negatively regulating *TaAGPL1*-*1D* gene expression.

### Indirect regulation of *TaPDIL1*-*2* gene on expression of *TaAGPL1*-*1D* gene

Most of PDIL proteins contain a KDEL at the C-terminus, and are localized in ER, where they combine with other protein substrates to form enzymatic complexes, in which their classical thioredoxin domains are responsible for interacting with substrates [30]. In the enzymatic complexes, PDIL proteins function as chaperones to bind to the unfolded or partially folded protein substrates by ascertaining their correct folding and preventing their aggregation [31]. Cereal endosperm storage proteins (e.g., gliadins and glutenins) have many disulfide bonds, which are bound to some PDIL proteins [32].

**Fig. 5:**
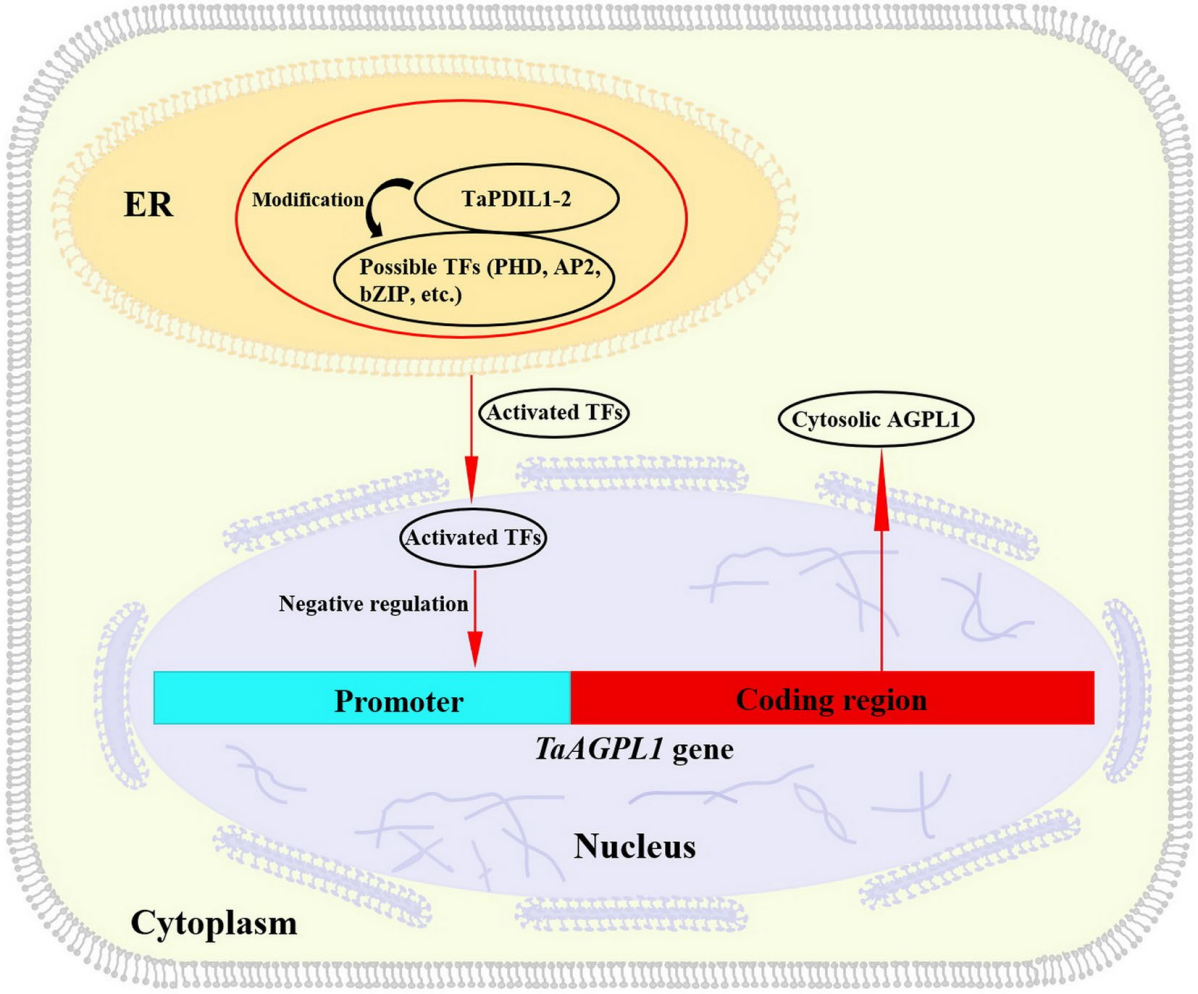
Schematic model of TaPDIL1-2 chaperone in a protein complex by modifying the folding of some transcription factors (TFs), which negatively regulates expression of the *TaAGPL1* gene

Transcription factors (TFs) function by binding to *cis*-acting elements preferentially located in the promoter region of the target genes [33], some TFs are also localized in ER to act as homo- or hetero-dimers with some chaperones (e.g. DnaK), where the cytoplasm-facing N-terminal regions are released by chaperones to be active TFs, and then, active TFs have been transferred from the ER into the nucleus, in which they activate the transcription of downstream genes [34]. For example, DRBEIII-1, a plant-specific TF in *Brassica napus*, was fused to a human PDIL protein to form a fusion protein complex (HDP), in which DREBIII-1 exhibited a highly soluble and biologically active form and HDP was confirmed to have the biological function of DRBEIII-1 [35]. Our sequence analysis indicated that the potential binding region was located from − 483 to − 1269 bp in the *TaAGPL1*-*1D* promoter containing the Auxin-responsive element and GCC-box-binding *cis*-acting elements (Additional file 8: Fig. S6). Previous studies have shown that a few PHD-, or AP2/ERF, bZIP-type TFs negatively regulate the expression of some starch biosynthesis genes [10, 36–40], and some members (e.g. bZIP28 and bZIP60) are localized in ER and possess the capacity to preferentially on the promoters of downstream target genes via binding to the above-mentioned *cis*-acting elements [41, 42]. Therefore, we speculated that functional TaPDIL1-2 protein could combine with TFs in ER to ascertain their correct folding to activate TFs, and then, active TFs are transferred into nucleus, in which they negatively regulate the expression of the *TaAGPL1*-*1D* gene. Based on these results, we propose one pathway for the TaPDIL1-2 chaperone to indirectly and negatively regulate the expression of the *TaAGPL1* gene in starch biosynthesis (Fig. 5).

## Conclusions

We isolated the promoter of *TaAGPL1-1DL* gene and constructed a wheat endosperm cDNA library. Through a yeast one-hybrid screening by using the promoter of *TaAGPL1*-*1DL* gene as bait, and wheat grain cDNA library as prey, we further identified a protein disulfide isomerase (TaPDIL1-2), which bound to the promoter of *TaAGPL1*-*1DL* gene. By using BSMV-VIGS method in field experiments, we furthermore found that *TaPDIL1*-*2* gene could negatively regulate the bread wheat starch biosynthesis.

## Supplementary information

**Additional file 1: Table S1.** The primer sequences used in this study.

**Additional file 2: Fig. S1.** Isolated sequence of the TaAGPL1-1D promoter from bread wheat cv. Zhoumai 18.

**Additional file 3: Fig. S2.** GUS histochemical staining and activity determination of the TaAGPL1-1DL promoter in *N*. *benthamiana* leaves.

**Additional file 4: Fig. S3.** The identified quality of wheat grains cDNA library.

**Additional file 5: Table S2.** The identified protein species via Y1H screening by using TaAGPL1-1D promoter as bait and wheat endosperm cDNA library as prey.

**Additional file 6: Fig. S4.** The open reading frame (ORF) of the isolated *TaPDIL1*-*2* gene.

**Additional file 7: Fig. S5** The partial cDNA sequence of three homoeologs of the *TaPDIL1*-*2* gene used for BSMV-VIGS experiment.

**Additional file 8: Fig. S6.** The main *cis*-acting elements in partial fragment (786 bp, from − 483 to − 1269 bp) of *TaAGPL1*-*1D* promoter.

## Data Availability

Please contact author for data requests.

## Abbreviations

AGPase: ADP-glucose pyrophosphorylase
BSMV-VIGS: barley stripe mosaic virus-induced gene-silencing
TaPDIL1-2: wheat disulfide isomerase 1-2 protein
G-1-P: glucose-1-phosphate
LSU or AGPL: large/modulatory subunits of AGPase
SSU or AGPS: small/catalytic subunits of AGPase
Y1H: yeast one-hybrid
IWGSC: International Wheat Genome Sequencing Consortium
qPCR: quantitative real-time PCR
GAPDH: glyceraldehyde 3-phosphate dehydrogenase
ANOVA: one-way analysis of variance
SD: standard deviation
ORF: open reading frame
TFs: transcription factors
ER: endoplasmic reticulum
