# Supplementary material for: PDIL1-2 can indirectly and negatively regulate expression of the AGPL1 gene in bread wheat
Source: Biol Res. 2019 Nov 7;52:56. doi: 10.1186/s40659-019-0263-2 (PMC6839113; doi:10.1186/s40659-019-0263-2)
Supplement: Supplementary file 1 — Additional file 1: Table S1. The primer sequences used in this study. [file 40659_2019_263_MOESM1_ESM.docx]

**Table S1 The primer sequences used in this study.**

| Function | Primer name | Primer sequence (5’-3’) | Sizes of amplified fragments (bp) |
| --- | --- | --- | --- |
| pCAMBIA1301-GUS-TaAGPL1 | pCAMBIA1301-Pro-1 F | TACGAATTCGAGCTCTCTTCTTCCCTGCATTTGAT | 1280 |
|  | pCAMBIA1301-Pro-1 R | TCAGATCTACCATGGATCGATGACATTCGCAGT |  |
|  | pCAMBIA1301-Pro-2 F | TACGAATTCGAGCTCGAAGAGGACGGATCTTGTTG | 881 |
|  | pCAMBIA1301-Pro-1 R | TCAGATCTACCATGGATCGATGACATTCGCAGT |  |
|  | pCAMBIA1301-Pro-3 F | TACGAATTCGAGCTCTCACATGTGCATCTTTCCTC | 494 |
|  | pCAMBIA1301-Pro-1 R | TCAGATCTACCATGGATCGATGACATTCGCAGT |  |
| Identification of positive colonies for Y1H | T7-F | TAATACGACTCACTATAGGG | > 500 |
|  | 3AD-R | AGATGGTGCACGATGCACAG |  |
| pAbAi-TaAGPL1 | pAbAi-Pro-1 F | CTTGAATTCGAGCTCTCTTCTTCCCTGCATTTGAT | 1280 |
|  | pAbAi-Pro-1 R | CCGGGTACCGAGCTCATCGATGACATTCGCAGT |  |
|  | pAbAi-Pro-2 F | CTTGAATTCGAGCTCGAAGAGGACGGATCTTGTTG | 881 |
|  | pAbAi-Pro-2 R | CCGGGTACCGAGCTCATCGATGACATTCGCAGT |  |
|  | pAbAi-Pro-3 F | CTTGAATTCGAGCTCTCACATGTGCATCTTTCCTC | 494 |
|  | pAbAi-Pro-3 R | CCGGGTACCGAGCTCATCGATGACATTCGCAGT |  |
| pGADT7-TaPDIL1-2 | TaPDIL F | GATTACGCTCATATGATGGCGATCTCCAAGGTC | 1539 |
|  | TaPDIL R | CATCTGCAGCTCGAGGAGCTCGTCCTTCAGAGG |  |
| BSMV-VIGS | VIGS-TaPDIL F | CCTTAATTAAATGCCAACCATCTCCCAC | 190 |
|  | VIGS-TaPDIL R | TATGCGGCCGCAAGAGCATGGCCTTGGGA |  |
| qPCR | qTaPDIL1-2 F | CAGTGGAGAGGCCATTGGTT | 120 |
|  | qTaPDIL 1-2 R | CAACTTTCGGGGTGCTGCTA |  |
|  | qTaAGPL1-1 F | GCCCCTGTTGGAGAGAGCCG | 157 |
|  | qTaAGPL1-1 R | TAGCAGGGTCGTCGATGGCG |  |
|  | qTaActin F | CCTCTCTTAGCACTTTCCAGCA | 101 |
|  | qTaActin R | GTAAGTCCCCTTCACCGACTC |  |
|  | qTaGAPDH F | GGTGAAGGACTCCAAGACCC | 179 |
|  | qTaGAPDH R | CCGGTGGACTCAACAACGTA |  |
