# Supplementary material for: PDIL1-2 can indirectly and negatively regulate expression of the AGPL1 gene in bread wheat
Source: Biol Res. 2019 Nov 7;52:56. doi: 10.1186/s40659-019-0263-2 (PMC6839113; doi:10.1186/s40659-019-0263-2)
Supplement: Supplementary file 2 — Additional file 2: Fig. S1. Isolated sequence of the TaAGPL1-1D promoter from bread wheat cv. Zhoumai 18. [file 40659_2019_263_MOESM2_ESM.docx]

TaAGPL1 CS TCTTCTTCCCTGCATTTGATTGATCCGTCGCTTGCCCGGTCGCCCGTCGATCCGTTTACCCGCCGTCCGCCCGTCCCCGG 80

TaAGPL1-1DL TCTTCTTCCCTGCATTTGATTGATCCGTCGCTTGCCCGGTCGCCCGTCGATCCGTTTACCCGCCGTCCGCCCGTCCCCGG 80

TaAGPL1 CS CAGCCGCAGCCGCACCGCAGGTAACAACACATTTCACCCCCTCCCTCCCCCCTTGCCGATTCATTAATTTGCATTCCGGT 160

TaAGPL1-1DL CAGCCGCAGCCGCACCGCAGGTAACAACACATTTCACCCCCTCCCTCCCCCCTTGCCGATTCATTAATTTGCATTCCGGT 160

TaAGPL1 CS GGCGCAAAGGTGGCGGGCGGGCGCTGCCTGTTCCTCCCGGCCGGACTCCGCCGCGCATTAATGCCGCCCCATTCCGACGC 240

TaAGPL1-1DL GGCGCAAAGGTGGCGGGCGGGCGCTGCCTGTTCCTCCCGGCCGGACTCCGCCGCGCATTAATGCCGCCCCATTCCGACGC 240

TaAGPL1 CS GGGCGGGCGGGGCACGGGAGGGGCGGTGGTGGACCGATTGGTCGGGCGGCGGTGCGTACCGGTGGGGGCGCCCGTGAGGT 320

TaAGPL1-1DL GGGCGGGCGGGGCACGGGAGGGGCGGTGGTGGACCGATTGGTCGGGCGGCGGTGCGTACCGGTGGGGGCGCCCGTGAGGT 320

TaAGPL1 CS GAGTGAGTGAGTGACACCGCGCGCGCGGCGCCGGCGGCGTGGTTGGGGCAAAACTGTCGACCGGCCGCTGGCGAGGAGGG 400

TaAGPL1-1DL GAGTGAGTGAGTGACACCGCGCGCGCGGCGCCGGCGGCGTGGTTGGGGCAAAACTGTCGACCGGCCGCTGGCGAGGAGGG 400

TaAGPL1 CS AAGAGGACGGATCTTGTTGCCAACTTAATTTCAGGATCACGGCCGCTCGGTTTCTCATTAACACTAGCAGTACCATGCTG 480

TaAGPL1-1DL AAGAGGACGGATCTTGTTGCCAACTTAATTTCAGGATCACGGCCGCTCGGTTTCTCATTAACACTAGCAGTACCATGCTG 480

TaAGPL1 CS ATTTATTTTCCTCCTGTCTGAATTAACTGTTGCCAACGACTGAATATCATCACTTCACTCATTTCATTCGTCGCTGTAAA 560

TaAGPL1-1DL ATTTATTTTCCTCCTGTCTGAATTAACTGTTGCCAACGACTGAATATCATCACTTCACTCATTTCATTCGTCGCTGTAAA 560

TaAGPL1 CS AGGGAGGAGGCTCGCCGGTGATTGGGGCGAGTCCTCCTTTCCTTTCCTCGATCTCTACTTGTGCCCAGTGGTGATTGCAG 640

TaAGPL1-1DL AGGGAGGAGGCTCGCCGGTGATTGGGGCGAGTCCTCCTTTCCTTTCCTCGATCTCTACTTGTGCCCAGTGGTGATTGCAG 640

TaAGPL1 CS GAGCATTTGTTTTGCGTTCCCGACGACAGGAAAATCGAGTCCTCCTTGGATCCTGCGCGCCCTGCTAAATTTTCTTCAAA 720

TaAGPL1-1DL GAGCATTTGTTTTGCGTTCCCGACGACAGGAAAATCGAGTCCTCCTTGGATCCTGCGCGCCCTGCTAAATTTTCTTCAAA 720

TaAGPL1 CS TATTGCGGCTTCTAGGCTCTGACCAGCTCGGTTTCATCATCACCGCTGCCTTAAAAACAGCACCACTCACATGTGCATCT 800

TaAGPL1-1DL TATTGCGGCTTCTAGGCTCTGACCAGCTCGGTTTCATCATCGCCGCTGCCTTAAAAACAGCACCACTCACATGTGCATCT 800

TaAGPL1 CS TTCCTCGTGTCCACAACAAGTTCACATGTGCAGTGAAATCCTCGTGCAAGTCTCCGTTTCCACTTGCAAAGTTCATGGGG 880

TaAGPL1-1DL TTCCTCGTGTCCACAACAAGTTCACATGTGCAGTGAAATCCTCGTGCAAGTCTCCGTTTCCACTTGCAAAGTTCATGGGG 880

TaAGPL1 CS ATATTCTGCCTTGCTTCCCTTTGGGTTCAGAAGAGGAAACAGCGAAGGAAGAACGCCAGCCACAAGATCAGAGGATCACC 960

TaAGPL1-1DL ATATTCTGCCTTGCTTCCCTTTGAGTTCAGAAGAGGAAACAGCGAAGGAAGAACGCCAGCCACAAGATCAGAGGATCACC 960

TaAGPL1 CS TCCTGATTCACGTGACCCTGCCCTGAAAAGTCACAAAGTCCTCGTCACCTTTCCCACTCTTCCACTAGTCCCTTTTCCCA 1040

TaAGPL1-1DL TCCTGATTCACGTGACCCTGCCCTGAAAAGTCACAAAGTCCTCGTCACCTTTCCCACTCTTCCACTAGTCCCTTTTCCCA 1040

TaAGPL1 CS TGATCATTGTTCTGAAGTATACTGAGCTTTACAGAGGGCCGTGACCCGTGAGAGAGGGGAAAAAGCCCGTGCTCTTTTTT 1120

TaAGPL1-1DL TGATCATTGTTCTGAAGTATACTGAGCTTTACAGAGGGCCGTGACCCGTGAGAGAGGGGAAAAAGCCCGTGCTCTTTTTT 1120

TaAGPL1 CS GCTCGACCTGTAAATTCATCAGTGATTTTGGGCCAAGTCATGTGCCTTCCATGCTCCAATCTAGTCTACCAGTTAATGGA 1200

TaAGPL1-1DL GCTCGACCTGTAAATTCATCAGTGATTTTGGGCCAAGTCATGTGCCTTCCATGCTCCAATCTAGTCTACCAGTTAATGGA 1200

TaAGPL1 CS TTCTGAAGATTCCATGGTTGCTGATCGTTCTCAACTTCTCATCTCGTTGCAGTTGCAGGTGGACTGCGAATGTCATCGAT 1280

TaAGPL1-1DL TTCTGAAGATTCCATGGTTGCTGATCGTTCTCAACTTCTCATCTTGTTGCAGTTGCAGGTGGACTGCGAATGTCATCGAT 1280

**Fig. S1** **Isolated sequence of the *TaAGPL1-1D* promoter from bread wheat cv. Zhoumai 18.** White pairs signed with white color are defined as different bases between bread wheat cv. Zhoumai 18 and cv. Chinese Spring (CS) derived from IWGSC database. The amplified *TaAGPL1-1D* promoter was 1280 bp, which has high similarity (99.8%) with that of cv. CS.
