# Supplementary material for: PDIL1-2 can indirectly and negatively regulate expression of the AGPL1 gene in bread wheat
Source: Biol Res. 2019 Nov 7;52:56. doi: 10.1186/s40659-019-0263-2 (PMC6839113; doi:10.1186/s40659-019-0263-2)
Supplement: Supplementary file 3 — Additional file 3: Fig. S2. GUS histochemical staining and activity determination of the TaAGPL1-1DL promoter in N. benthamiana leaves. [file 40659_2019_263_MOESM3_ESM.docx]

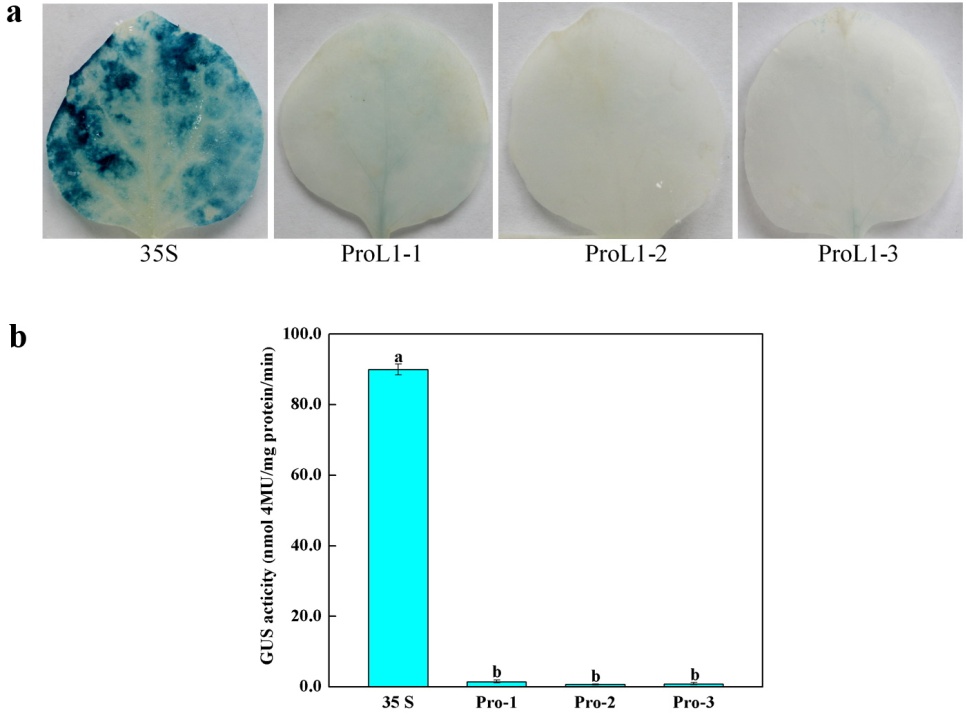


**Fig. S2 GUS histochemical staining and activity determination of the *TaAGPL1-1DL* promoter in *N. Benthamiana* leaves.** a, GUS histochemical staining of *N. Benthamiana* leaves. 35 S represents CaMV35S promoter; b, GUS activity determination of *TaAGPL1-1D* in *N. Benthamiana* leaves.
