# Supplementary material for: PDIL1-2 can indirectly and negatively regulate expression of the AGPL1 gene in bread wheat
Source: Biol Res. 2019 Nov 7;52:56. doi: 10.1186/s40659-019-0263-2 (PMC6839113; doi:10.1186/s40659-019-0263-2)
Supplement: Supplementary file 4 — Additional file 4: Fig. S3. The identified quality of wheat grains cDNA library. [file 40659_2019_263_MOESM4_ESM.docx]

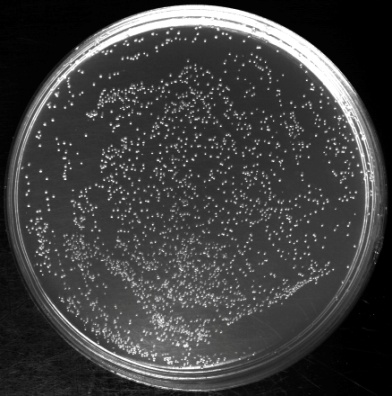


**a**

**750 bp**

**750 bp**

**750 bp**

**750 bp**


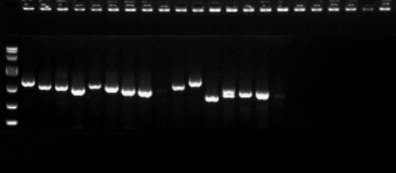

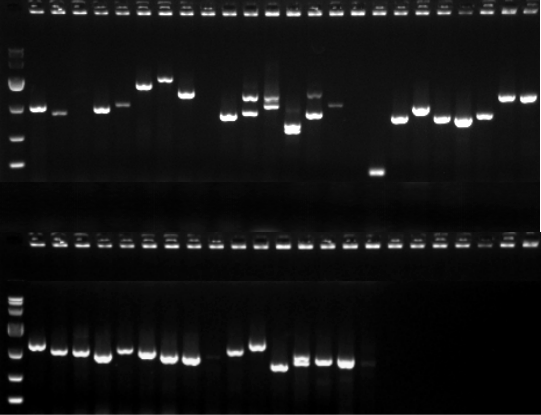

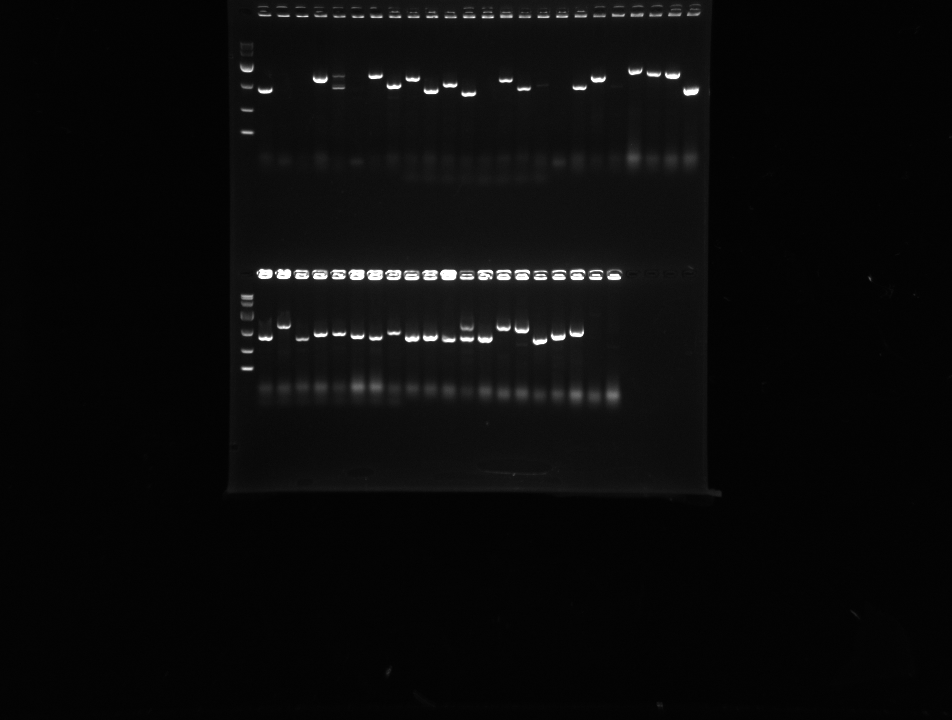

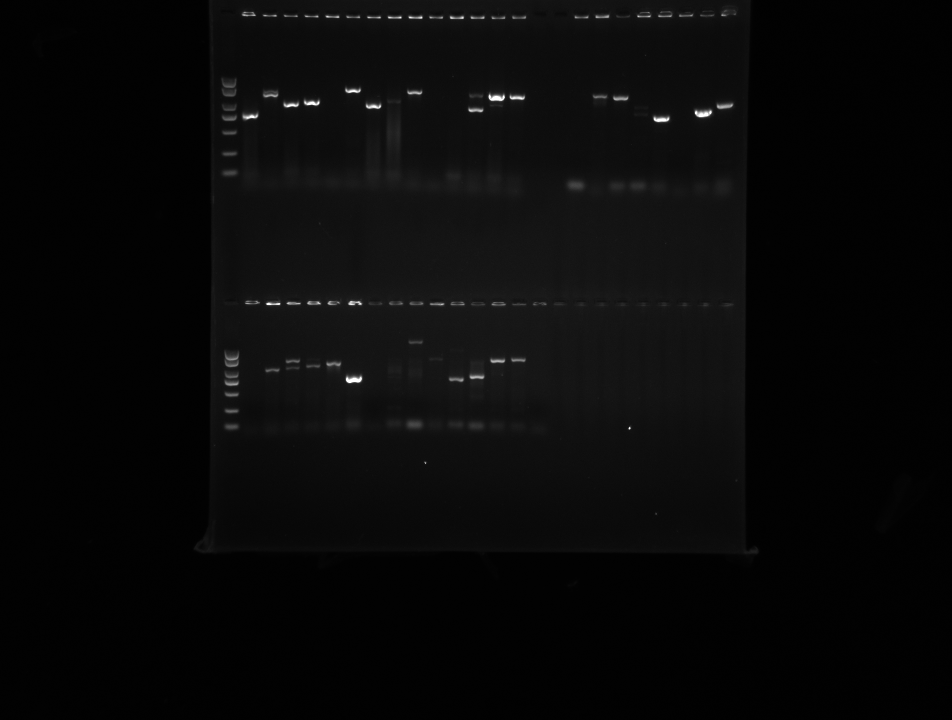


M

**M**

**M**

**b**

**Fig. S3** **The identified quality of wheat grains cDNA library.** Wheat grains were sampled at 5, 10, 15, 20, 25, 30, and 35 days after anthesis, and were mixed and used for cDNA library. a, The growth status of cDNA library in SD/-Leu,-Ura medium; b, Fragment sizes in cDNA library of wheat grains.
