# Supplementary material for: PDIL1-2 can indirectly and negatively regulate expression of the AGPL1 gene in bread wheat
Source: Biol Res. 2019 Nov 7;52:56. doi: 10.1186/s40659-019-0263-2 (PMC6839113; doi:10.1186/s40659-019-0263-2)
Supplement: Supplementary file 5 — Additional file 5: Table S2. The identified protein species via Y1H screening by using TaAGPL1-1D promoter as bait and wheat endosperm cDNA library as prey. [file 40659_2019_263_MOESM5_ESM.docx]

**Table S2 The identified protein species *via* Y1H screening by using *TaAGPL1-1D* promoter as bait and wheat endosperm cDNA library as prey.**

| Accession no. (NCBI) | Description | Species |
| --- | --- | --- |
| EMS51411.1 | 40S ribosomal protein S26-3 | *Triticum urartu* |
| AAV91999.1 | Low molecular weight glutenin | *Triticum aestivum* |
| XP-020180381.1 | Avenin-3-like | *Aegilops tauschii* |
| APU92363.1 | Alpha-gliadin storage protein | *Triticum spelta* |
| XP-020172867.1 | Peptidyl-prolyl cis-trans isomerase | *Aegilops tauschii* |
| AB933344.1 | Protein disulfide isomerase family protein 1-2 | *Triticum aestivum* |
| CAA42453.1 | CM 17 protein precursor | *Triticum aestivum* |
| XP-020173542.1 | Elongation factor 1-alpha | *Aegilops tauschii* |
| CAH04987.1 | Type 1 non-specific lipid transfer protein precursor | *Triticum aestivum* |
| XP-020193272.1 | FH protein interacting protein FIP2 | *Aegilops tauschii* |
| CDJ26291.1 | Unnamed protein product | *Triticum aestivum* |
| XP-010232477.2 | ADP-ribosylation factor 2 | *Brachypodium distachyon* |
| CAA54191.1 | Type V Thionin | *Triticum aestivum* |
| XP-020195452.1 | 60S ribosomal protein L19-1-like isoform X1 | *Aegilops tauschii* |
| AGU91656.1 | LMW-m glutenin subunit 2 | *Triticum aestivum* |
| EMS51899.1 | Hypothetical protein TRIUR3_17976 | *Triticum urartu* |
| XP-020175915.1 | Ubiquitin-conjugating enzyme E2 28 | *Aegilops tauschii* |
| EMS64771.1 | Hypothetical protein TRIUR3_26004 | *Triticum urartu* |
| CAH10199.1 | Pinb Puroindoline-b | *Triticum aestivum* |
| XP-020181310.1 | DnaJ protein ERDJ3B isoform X1 | *Aegilops tauschii* |
| AAL67139.1 | Thioredoxin H | *Triticum aestivum* |
| EMS53525.1 | Putative serine incorporator | *Triticum urartu* |
| EMS68426.1 | Heat shock cognate 70 kDa protein 4 | *Triticum urartu* |
| KJ461851.1 | Cultivar Chinese Spring AHL protein (AHL2) gene | *Triticum aestivum* |
| XP-020158648.1 | Early nodulin-93-like | *Aegilops tauschii* |
| AFQ60540.1 | Alpha purothionin | *Triticum aestivum* |
| EMS65097.1 | Hypothetical protein TRIUR3_16979 | *Triticum urartu* |
| XP-020152460.1 | Alpha-amylase inhibitor 0.28 | *Aegilops tauschii* |
| EMS62569.1 | Avenin-3 | *Triticum urartu* |
| CAQ43070.2 | Putative puroindoline b protein | *Triticum macha* |
| AEG74431.1 | Alpha-gliadin | *Triticum compactum* |
| XP-020191910.1 | triphosphate tunel metalloenzyme 3-like | *Aegilops tauschii* |
| AFB35199.1 | 9-7 alpha-gliadin (gli-2) gene | *Triticum aestivum* |
| XP-020182679.1 | PX domain-containing protein EREL1 | *Aegilops tauschii* |
| APU92371.1 | Alpha-gliadin storage protein | *Triticum spelta* |
| XP-020185218.1 | Calcineurin subunit B-like | *Aegilops tauschii* |
| ABN71646.1 | Truncated high molecular weight glutenin subunit 1By9 | *Triticum aestivum* |
| XP-020180332.1 | Cullin-1 isoform X2 | *Aegilops tauschii* |
| XP-020168539.1 | Serpin-Z1B | *Aegilops tauschii* |

Notes: NCBI database is used to identify the protein species from Y1H.
