# Supplementary material for: PDIL1-2 can indirectly and negatively regulate expression of the AGPL1 gene in bread wheat
Source: Biol Res. 2019 Nov 7;52:56. doi: 10.1186/s40659-019-0263-2 (PMC6839113; doi:10.1186/s40659-019-0263-2)
Supplement: Supplementary file 6 — Additional file 6: Fig. S4. The open reading frame (ORF) of the isolated TaPDIL1-2 gene. [file 40659_2019_263_MOESM6_ESM.docx]

TaPDIL CS ATGGCGATCTGCAAGGTCTGGATCTCGCTGCTGCTCGCGCTCGCCGTCGTCCTGTCCGCCCCGGCGGCCAGGGCGGAGGAGGCTG 85

TaPDIL 1-2 ATGGCGATCTGCAAGGTCTGGATCTCGCTGCTGCTCGCGCTCGCCGTCGTCCTGTCCGCCCCGGCGGCCAGGGCGGAGGAGGCTG 85

TaPDIL CS CCGCCGCCGAGGAGGCCGCCGCGGCCCCCGAGGCCGTGCTCACCCTGCACGCCGACAACTTCGACGACGCCATCGCCAAGCACCC 170

TaPDIL 1-2 CCGCCGCCGAGGAGGCCGCCGCGGCCCCCGAGGCCGTGCTCACCCTGCACGCCGACAACTTCGACGACGCCATCGCCAAGCACCC 170

TaPDIL CS CTTCATCCTCGTCGAGTTCTACGCCCCATGGTGTGGACACTGCAAGAGCTTGGCACCTGAGTATGAGAAGGCGGCCCAACTGTTG 255

TaPDIL 1-2 CTTCATCCTCGTCGAGTTCTACGCCCCATGGTGTGGACACTGCAAGAGCTTGGCACCTGAGTATGAGAAGGCGGCCCAACTGTTG 255

TaPDIL CS AGCAAGCACGACCCAGCGATTGTTCTCGCTAAAGTTGATGCCAACGATGAGAAGAACAAGCCGCTTGCGGGCAAGTACGAGGTCC 340

TaPDIL 1-2 AGCAAGCACGACCCAGCGATTGTTCTCGCTAAAGTTGATGCCAACGATGAGAAGAACAAGCCGCTTGCGGGCAAGTACGAGGTCC 340

TaPDIL CS AGGGCTTCCCTACCCTCAAGATCTTCAGGAACGGAGGAAAGAACATTCAGGAATACAAGGGCCCCAGGGAGGCTGAGGGAATTGT 425

TaPDIL 1-2 AGGGCTTCCCTACCCTCAAGATCTTCAGGAACGGAGGAAAGAACATTCAGGAATACAAGGGCCCCAGGGAGGCTGAGGGAATTGT 425

TaPDIL CS TGAGTACTTGAAGAAGCAGGTTGGCCCTGCTTCCAAGGAGATCAAGGCACCTGAAGATGCCACTTACCTCGAAGACGGCAAGATC 510

TaPDIL 1-2 TGAGTACTTGAAGAAGCAGGTTGGCCCTGCTTCCAAGGAGATCAAGGCACCTGAAGATGCCACTTACCTCGAAGACGGCAAGATC 510

TaPDIL CS CACATTGTTGGTGTCTTCACGGAATTCAGCGGCACTGAGTTTACAAACTTCCTTGAGGTTGCTGAGAAGCTGAGGTCTGATTATG 595

TaPDIL 1-2 CACATTGTTGGTGTCTTCACGGAATTCAGCGGCACTGAGTTTACAAACTTCCTTGAGGTTGCTGAGAAGCTGAGGTCTGATTATG 595

TaPDIL CS ACTTTGGCCACACCGTGCATGCCAACCATCTCCCACGTGGTGATGCCGCAGTGGAGAGGCCATTGGTTAGGCTATTCAAGCCATT 680

TaPDIL 1-2 ACTTTGGCCACACCGTGCATGCCAACCATCTCCCACGTGGTGATGCCGCAGTGGAGAGGCCATTGGTTAGGCTATTCAAGCCATT 680

TaPDIL CS TGATGAGCTCGTTGTTGACAGCAAGGATTTTGATGTTTCTGCTTTGGAGAAATTCATTGAGGCTAGCAGCACCCCGAAAGTTGTT 765

TaPDIL 1-2 TGATGAGCTCGTTGTTGACAGCAAGGATTTTGATGTTTCTGCTTTGGAGAAATTCATTGAGGCTAGCAGCACCCCGAAAGTTGTT 765

TaPDIL CS ACTTTTGACAAGAACCCTGACAACCATCCTTACCTCTTGAAATTCTTCCAGAGCAATGCTCCCAAGGCCATGCTCTTTTTGAACT 850

TaPDIL 1-2 ACTTTTGACAAGAACCCTGACAACCATCCTTACCTCTTGAAATTCTTCCAGAGCAATGCTCCCAAGGCCATGCTCTTTTTGAACT 850

TaPDIL CS TCTCCACTGGACCGTTTGAGTCCTTCAAGAAAGCCTACTATGGTGCTGTAGAGGAGTTCAGCGGCAAGGATGTCAAGTTCCTAAT 935

TaPDIL 1-2 TCTCCACTGGACCGTTTGAGTCCTTCAAGAAAGCCTACTATGGTGCTGTAGAGGAGTTCAGCGGCAAGGATGTCAAGTTCCTAAT 935

TaPDIL CS TGGTGACATTGAAGCGAGCCAAGGCGCTTTCCAGTACTTCGGGCTGAAAGAGGATCAGGCACCACTTATCCTCATTCAAGACAGT 1020

TaPDIL 1-2 TGGTGACATTGAAGCGAGCCAAGGCGCTTTCCAGTACTTCGGGCTGAAAGAGGATCAGGCACCACTTATCCTCATTCAAGACAGT 1020

TaPDIL CS GACTCGAAGAAGTTTTTGAAGGAACAGGTTGAGGCTGGCCAAATTGTTGCTTGGTTGAAGGATTACTTCGATGGCAAATTGACAC 1105

TaPDIL 1-2 GACTCGAAGAAGTTTTTGAAGGAACAGATTGAGGCTGGCCAAATTGTTGCTTGGTTGAAGGATTACTTCGATGGCAAATTGACAC 1105

TaPDIL CS CATTCAGGAAGTCCGAGCCTATTCCTGAGGCCAACAATGAGCCTGTTAAGGTAGTTGTGGCTGACAACGTTCACGACGTGGTCTT 1190

TaPDIL 1-2 CATTCAGGAAGTCCGAGCCTATTCCTGAGGCCAACAATGAGCCTGTTAAGGTAGTTGTGGCTGACAACGTTCACGACGTGGTCTT 1190

TaPDIL CS CAAATCTGGCAAAAATGTTCTTATTGAATTCTATGCGCCCTGGTGCGGACACTGCAAGAAGCTAGCACCCATCCTCGACGAGGCA 1275

TaPDIL 1-2 CAAATCTGGCAAAAATGTTCTTATTGAATTCTATGCGCCCTGGTGCGGACACTGCAAGAAGCTAGCACCCATCCTCGACGAGGCA 1275

TaPDIL CS GCTGCCACCCTTCAAAGCGAAGAGGACGTTGTGATCGCGAAGATGGACGCGACCGCGAATGACGTGCCCAGTGAGTTCGATGTCC 1360

TaPDIL 1-2 GCTGCCACCCTTCAAAGCGAAGAGGACGTTGTGATCGCGAAGATGGACGCGACCGCGAATGACGTGCCCAGTGAGTTCGATGTCC 1360

TaPDIL CS AGGGTTACCCCACCCTCTACTTCGTCACTCCCAGCGGAAAGAAGGTCTCCTACGAGGGCGGCAGGACGGCCGATGAGATTGTCGA 1445

TaPDIL 1-2 AGGGTTACCCCACCCTCTACTTCGTCACTCCCAGCGGAAAGAAGGTCTCCTACGAGGGCGGCAGGACGGCCGATGAGATTGTCGA 1445

TaPDIL CS CTACATCAAGAAGAACAAGGAGACTGCCGGGCAGGCGGCGACCGAGAAGGCGGCGGAACCGGCTGCCACGGAGCCTCTGAAGGAC 1530

TaPDIL 1-2 CTACATCAAGAAGAACAAGGAGACTGCCGGGCAGGCGGCGACCGAGAAGGCGGCGGAACCGGCTGCCACGGAGCCTCTGAAGGAC 1530

TaPDIL CS GAGCTCTGA 1539

TaPDIL 1-2 GAGCTCTGA 1539

**Fig. S4** **The open reading frame (ORF) of the isolated *TaPDIL1-2* gene.** This sequence was compared with that from cv. Chinese Spring (CS).
