# Supplementary material for: PDIL1-2 can indirectly and negatively regulate expression of the AGPL1 gene in bread wheat
Source: Biol Res. 2019 Nov 7;52:56. doi: 10.1186/s40659-019-0263-2 (PMC6839113; doi:10.1186/s40659-019-0263-2)
Supplement: Supplementary file 7 — Additional file 7: Fig. S5 The partial cDNA sequence of three homoeologs of the TaPDIL1-2 gene used for BSMV-VIGS experiment. [file 40659_2019_263_MOESM7_ESM.docx]

TaPDIL-4AL-CDS ATGGCGATCTCCAAGGTCTGGATCTCGCTGCTGCTCGCGCTCGCCGTCGTCCTGTCCGCCCCGGCGGCCAGGGCGGAGGAGGCCG 85

TaPDIL-4BS-CDS ATGGCGATCTGCAAGGTCTGGATCTCGCTGCTGCTCGCGCTCGCCGTCGTCCTGTCCGCCCCGGCGGCCAGGGCGGAGGAGGCTG 85

TaPDIL-4DS-CDS ATGGCGATCTGCAAGGCCTGGATCTCGCTGCTGCTCGCGCTCGCCGTCGTCCTGTCCGCCCCGGCGGCCAGGGCGGAGGAGGCCG 85

TaPDIL-4AL-CDS CCGCCGCCGAGGAGGCCGCCGCGGCCCCCGAGGCAGTGCTCACCCTGCACGCCGACAACTTCGACGACGCCATCGCCAAGCACCC 170

TaPDIL-4BS-CDS CCGCCGCCGAGGAGGCCGCCGCGGCCCCCGAGGCCGTGCTCACCCTGCACGCCGACAACTTCGACGACGCCATCGCCAAGCACCC 170

TaPDIL-4DS-CDS CCGCCGCCGCGGAGGAGGCTGCGGCCCCCGAGGCCGTGCTCACCCTGCACGCCGACAACTTCGACGACGCCATCGCCAAGCACCC 170

TaPDIL-4AL-CDS CTTCATCCTCGTCGAGTTCTACGCCCCATGGTGTGGACACTGCAAGAGCCTGGCACCAGAGTATGAGAAGGCGGCCCAACTGTTG 255

TaPDIL-4BS-CDS CTTCATCCTCGTCGAGTTCTACGCCCCATGGTGTGGACACTGCAAGAGCTTGGCACCTGAGTATGAGAAGGCGGCCCAACTGTTG 255

TaPDIL-4DS-CDS CTTCATCCTCGTCGAGTTCTACGCCCCATGGTGTGGACACTGCAAGAGCCTGGCACCTGAGTATGAGAAGGCGGCCCAACTGTTG 255

TaPDIL-4AL-CDS AGCAAGCACGACCCAGCGATCGTCCTTGCTAAGGTTGATGCCAACGATGAGAAGAACAAGCCGCTTGCGGGCAAGTACGAGGTCC 340

TaPDIL-4BS-CDS AGCAAGCACGACCCAGCGATTGTTCTCGCTAAAGTTGATGCCAACGATGAGAAGAACAAGCCGCTTGCGGGCAAGTACGAGGTCC 340

TaPDIL-4DS-CDS AGCAAGCACGACCCAGCGATTGTCCTTGCTAAGGTTGATGCCAACGATGAGAAGAACAAGCCGCTTGCGGGCAAGTACGAGGTCC 340

TaPDIL-4AL-CDS AGGGCTTCCCTACCCTCAAGATCTTCAGGAACGGCGGAAAGAACATCCAGGAATACAAGGGCCCCAGGGAGGCTGAGGGAATTGT 425

TaPDIL-4BS-CDS AGGGCTTCCCTACCCTCAAGATCTTCAGGAACGGAGGAAAGAACATTCAGGAATACAAGGGCCCCAGGGAGGCTGAGGGAATTGT 425

TaPDIL-4DS-CDS AGGGCTTCCCTACCCTCAAGATCTTCAGGAACGGGGGAAAGAACATCCAGGAATACAAGGGCCCCAGGGAGGCTGAGGGAATTGT 425

TaPDIL-4AL-CDS TGAGTACTTGAAGAAGCAGGTTGGCCCTGCTTCCAAGGAGATAAAGGCGCCTGAAGATGCCACTTACCTTGAAGACGGCAAGATC 510

TaPDIL-4BS-CDS TGAGTACTTGAAGAAGCAGGTTGGCCCTGCTTCCAAGGAGATCAAGGCACCTGAAGATGCCACTTACCTCGAAGACGGCAAGATC 510

TaPDIL-4DS-CDS CGAGTACTTGAAGAAGCAGGTTGGCCCTGCTTCCAAGGAGATCAAGGCACCTGAAGATGCCACTTACCTTGAAGACGGCAAGATC 510

TaPDIL-4AL-CDS CACATTGTTGGTGTCTTCACGGAATTCAGCGGCACTGAATTTACAAACTTCCTTGAGCTTGCTGAGAAGCTGCGGTCTGATTATG 595

TaPDIL-4BS-CDS CACATTGTTGGTGTCTTCACGGAATTCAGCGGCACTGAGTTTACAAACTTCCTTGAGGTTGCTGAGAAGCTGAGGTCTGATTATG 595

TaPDIL-4DS-CDS CACATTGTTGGTGTTTTCACTGAATTCAGCGGCACTGAGTTTACAAACTTCCTTGAGGTTGCTGAGAAGCTGAGGTCTGATTATG 595

TaPDIL-4AL-CDS ACTTTGGCCACACCGTGCATGCCAACCATCTCCCACGTGGTGATGCAGCAGTGGAGAGGCCATTGGTTAGGCTATTCAAGCCATT 680

TaPDIL-4BS-CDS ACTTTGGCCACACCGTGCATGCCAACCATCTCCCACGTGGTGATGCCGCAGTGGAGAGGCCATTGGTTAGGCTATTCAAGCCATT 680

TaPDIL-4DS-CDS ACTTTGGCCACACCGTGCATGCCAACCATCTCCCACGTGGTGATGCAGCAGTGGAGAGGCCATTGGTTAGGCTATTCAAGCCATT 680

TaPDIL-4AL-CDS TGATGAGCTCGTTGTTGACAGCAAGGATTTTGATGTTTCTGCTTTGGAGAAATTCATTGATGCTAGCAGCACCCCGAAAGTTGTT 765

TaPDIL-4BS-CDS TGATGAGCTCGTTGTTGACAGCAAGGATTTTGATGTTTCTGCTTTGGAGAAATTCATTGAGGCTAGCAGCACCCCGAAAGTTGTT 765

TaPDIL-4DS-CDS TGATGAGCTCGTTGTTGACAGCAAGGATTTTGATGTTTCTGCTTTGGAGAAATTCATTGATGCTAGCAGCACCCCGAAAGTTGTT 765

TaPDIL-4AL-CDS ACTTTTGACAAGAACCCTGACAACCATCCTTACCTCTTGAAATACTTTCAGAGCAATGCTCCCAAGGCCATGCTCTTTTTGAACT 850

TaPDIL-4BS-CDS ACTTTTGACAAGAACCCTGACAACCATCCTTACCTCTTGAAATTCTTCCAGAGCAATGCTCCCAAGGCCATGCTCTTTTTGAACT 850

TaPDIL-4DS-CDS ACTTTTGACAAGAACCCTGACAACCATCCTTACCTCCTGAAATTCTTCCAGACCAATGCTCCCAAGGCCATGCTCTTTTTGAACT 850

TaPDIL-4AL-CDS TCTCCACTGGACCGTTTGAGTCCTTCAAATCAGCCTACTATGGTGCTGTAGAGGAGTTCAGTGGCAAGGATGTGAAGTTCCTTAT 935

TaPDIL-4BS-CDS TCTCCACTGGACCGTTTGAGTCCTTCAAGAAAGCCTACTATGGTGCTGTAGAGGAGTTCAGCGGCAAGGATGTCAAGTTCCTAAT 935

TaPDIL-4DS-CDS TCTCCACTGGACCGTTTGAGTCCTTCAAATCAGCCTACTATGGTGCTGTAGAGGAGTTCAGTGGCAAGGATGTGAAGTTCCTTAT 935

TaPDIL-4AL-CDS TGGTGACATTGAAGCGAGCCAAGGCGCCTTCCAGTACTTTGGCCTGAAAGAGGATCAGGCACCACTTATCCTCATTCAAGACAGT 1020

TaPDIL-4BS-CDS TGGTGACATTGAAGCGAGCCAAGGCGCTTTCCAGTACTTCGGGCTGAAAGAGGATCAGGCACCACTTATCCTCATTCAAGACAGT 1020

TaPDIL-4DS-CDS TGGTGACATTGAAGCGAGCCAAGGCGCTTTCCAGTACTTCGGGCTGAAAGAGGATCAGGCACCACTGATCCTCATTCAAGACAGT 1020

TaPDIL-4AL-CDS GACTCGAAGAAGTTTTTGAAGGAACAGGTTGAGGCTGGCCAAATTGTTGCTTGGTTGAAGGATTACTTTGATGGCAAATTGACAC 1105

TaPDIL-4BS-CDS GACTCGAAGAAGTTTTTGAAGGAACAGGTTGAGGCTGGCCAAATTGTTGCTTGGTTGAAGGATTACTTCGATGGCAAATTGACAC 1105

TaPDIL-4DS-CDS GACTCGAAGAAGTTTTTGAAGGAACAGGTTGAGGCTGGCCAAATTGTTGCTTGGTTGAAGGATTACTTTGATGGCAAATTGACAC 1105

TaPDIL-4AL-CDS CATTCAGGAAGTCCGAGCCTATTCCTGAGGCCAACAATGAGCCTGTGAAGGTAGTTGTGGCTGACAACATTCACGACGTGGTCTT 1190

TaPDIL-4BS-CDS CATTCAGGAAGTCCGAGCCTATTCCTGAGGCCAACAATGAGCCTGTTAAGGTAGTTGTGGCTGACAACGTTCACGACGTGGTCTT 1190

TaPDIL-4DS-CDS CATTCAGGAAGTCTGAGCCTATTCCTGAGGCCAACAATGAGCCTGTTAAGGTAGTTGTGGCTGACAACGTTCACGACGTGGTCTT 1190

TaPDIL-4AL-CDS CAAATCTGGCAAAAATGTTCTTATCGAGTTCTATGCACCCTGGTGCGGACACTGCAAGAAGCTAGCACCCATCCTCGACGAGGCA 1275

TaPDIL-4BS-CDS CAAATCTGGCAAAAATGTTCTTATTGAATTCTATGCGCCCTGGTGCGGACACTGCAAGAAGCTAGCACCCATCCTCGACGAGGCA 1275

TaPDIL-4DS-CDS CAAATCTGGCAAAAATGTTCTTATCGAGTTCTATGCACCCTGGTGCGGACACTGCAAGAAGCTAGCACCCATCCTCGACGAGGCA 1275

TaPDIL-4AL-CDS GCTGCCACCCTTCAAAGCGAAGAGGACGTTGTGATTGCCAAGATAGACGCTACCGCGAATGACGTTCCCGGCGAGTTTGATGTCC 1360

TaPDIL-4BS-CDS GCTGCCACCCTTCAAAGCGAAGAGGACGTTGTGATCGCGAAGATGGACGCGACCGCGAATGACGTGCCCAGTGAGTTCGATGTCC 1360

TaPDIL-4DS-CDS GCTGCCACCCTTCAAAGTGAAGAGGACGTTGTGATCGCGAAGATGGACGCGACCGCGAATGACGTGCCCAGTGAGTTCGATGTCC 1360

TaPDIL-4AL-CDS AGGGTTACCCCACCCTCTACTTCGTCACTCCTAGCGGGAAGAAGGTCTCGTATGAGGGCGGCAGGACGGCCGACGAGATTGTCGA 1445

TaPDIL-4BS-CDS AGGGTTACCCCACCCTCTACTTCGTCACTCCCAGCGGAAAGAAGGTCTCCTACGAGGGCGGCAGGACGGCCGATGAGATTGTCGA 1445

TaPDIL-4DS-CDS AGGGTTACCCCACCCTGTACTTCGTCACTCCCAGCGGGAAGAAGGTCTCGTACGAGGGCGGCAGGACGGCCGACGAGATCGTAGA 1445

TaPDIL-4AL-CDS CTACATCAAGAAGAACAAGGAGACTGCCGGGCAGGCGGCAGCGGCGGCGACCGAGAAGGCAGCTGAACCGGCTGCCACCGAGCCT 1530

TaPDIL-4BS-CDS CTACATCAAGAAGAACAAGGAGACTGCCGGGCA.........GGCGGCGACCGAGAAGGCGGCGGAACCGGCTGCCACGGAGCCT 1521

TaPDIL-4DS-CDS CTACATCAAGAAGAACAAGGAGACTGCTGGGCAGGCGGCTGCGGCGGACACCGAGAAGGCGGCGGAACCGGCTGCCACCGAGCCT 1530

TaPDIL-4AL-CDS CTGAAGGACGAGCTCTGA 1548

TaPDIL-4BS-CDS CTGAAGGACGAGCTCTGA 1539

TaPDIL-4DS-CDS CTGAAGGACGAGCTCTGA 1548

**Fig. S5 The partial cDNA sequence of three homoeologs of the *TaPDIL1-2* gene used for BSMV*-*VIGS experiment.** The fragments used for VIGS silencing experiment have been underlined with red color.
