# Supplementary material for: PDIL1-2 can indirectly and negatively regulate expression of the AGPL1 gene in bread wheat
Source: Biol Res. 2019 Nov 7;52:56. doi: 10.1186/s40659-019-0263-2 (PMC6839113; doi:10.1186/s40659-019-0263-2)
Supplement: Supplementary file 8 — Additional file 8: Fig. S6. The main cis-acting elements in partial fragment (786 bp, from − 483 to − 1269 bp) of TaAGPL1-1D promoter. [file 40659_2019_263_MOESM8_ESM.doc]

TCTTCTTCCCTGCATTTGATTGATCCGTCGCTTGCCCGGTCGCCCGTCGATCCGTTTACCCGCCGTCCGCCCGTCCCCGGCAGCCGCAGCCGCACCGCAGGTAACAACACATTTCACCCCCTCCCTCCCCCCTTGCCGATTCATTAATTTGCATTCCGGTGGCGCAAAGGTGGCGGGCGGGCGCTGCCTGTTCCTCCCGGCCGGACTCCGCCGCGCATTAATGCCGCCCCATTCCGACGCGGGCGGGCGGGGCACGGGAGGGGCGGTGGTGGACCGATTGGTCGGGCGGCGGTGCGTACCGGTGGGGGCGCCCGTGAGGTGAGTGAGTGAGTGACACCGCGCGCGCGGCGCCGGCGGCGTGGTTGGGGCAAAACTGTCGACCGGCCGCTGGCGAGGAGGGAAGAGGACGGATCTTGTTGCCAACTTAATTTCAGGATCACGGCCGCTCGGTTTCTCATTAACACTAGCAGTACCATGCTGATTTATTTTCCTCCTGTCTGAATTAACTGTTGCCAACGACTGAATATCATCACTTCACTCATTTCATTCGTCGCTGTAAAAGGGAGGAGGCTCGCCGGTGATTGGGGCGAGTCCTCCTTTCCTTTCCTCGATCTCTACTTGTGCCCAGTGGTGATTGCAGGAGCATTTGTTTTGCGTTCCCGACGACAGGAAAATCGAGTCCTCCTTGGATCCTGCGCGCCCTGCTAAATTTTCTTCAAATATTGCGGCTTCTAGGCTCTGACCAGCTCGGTTTCATCATCGCCGCTGCCTTAAAAACAGCACCAC

**Fig. S6 The main *cis*-acting elements in partial fragment (786 bp, from *-*483 to *-*1269 bp) of *TaAGPL1-1D* promoter.** The sequences marked with green and yellow colors represent ARE (TGTCNN) and GCC-box (GCCGCC), which preferentially bind with plant PHD-finger and ERF-type transcription factors, respectively.
